# Initial rigid response and softening transition of highly stretchable kirigami sheet materials

**DOI:** 10.1038/srep24758

**Published:** 2016-04-27

**Authors:** Midori Isobe, Ko Okumura

**Affiliations:** 1 Department of Physics and Soft Matter Center, Ochanomizu University, 2–1–1, Otsuka, Bunkyo-ku, Tokyo 112-8610, Japan

## Abstract

We study, experimentally and theoretically, the mechanical response of sheet materials on which line cracks or cuts are arranged in a simple pattern. Such sheet materials, often called kirigami (the Japanese words, kiri and gami, stand for cut and paper, respectively), demonstrate a unique mechanical response promising for various engineering applications such as stretchable batteries: kirigami sheets possess a mechanical regime in which sheets are highly stretchable and very soft compared with the original sheets without line cracks, by virtue of out-of-plane deformation. However, this regime starts after a transition from an initial stiff regime governed by in-plane deformation. In other words, the softness of the kirigami structure emerges as a result of a transition from the two-dimensional to three-dimensional deformation, i.e., from stretching to bending. We clarify the physical origins of the transition and mechanical regimes, which are revealed to be governed by simple scaling laws. The results could be useful for controlling and designing the mechanical response of sheet materials including cell sheets for medical regeneration and relevant to the development of materials with tunable stiffness and mechanical force sensors.

Sheet materials, such as paper, plastic film, and metal foil, are a familiar form of materials and useful in daily life, for example, for wrapping. However, their unique modes of mechanical responses are highly nontrivial and have actively been studied mainly from fundamental points of view, which includes crumpling of paper^1^,^2^, pleating of paper (Miura-ori or Origami)^3^,^4^,^5^, creasing of elastomer films^6^, wrinkling of thin sheets^7^,^8^, and twisting of ribbons^9^. Quite recently, it has been shown that such peculiar mechanical responses of sheet materials are also promising for engineering applications, such as foldable actuators^10^, self-folding shape-memory composites^11^, stretchable lithium-ion batteries^12^, stretchable electrodes^13^ stretchable graphens^14^,^15^, and integrated solar tracking^16^. One of the key factors in these quite recent engineering applications is the introduction of many cuts into sheet materials, often called the kirigami approach in recent papers. In principle, this approach allows us to design and control the elastic properties of sheet materials in a highly flexible manner. In fact, supermarkets in Japan often distribute one who buys bottles of wine with sheets of paper perforated with regularly arranged cuts to protect the bottles (see Fig. 1(a)). Similarly, a Japanese design factory produces “airvase” (Fig. 1(b)) sold in museum shops worldwide. Figure 1(c) demonstrates a paper with similar cuts in planer tension.

However, any simple relations between the mechanical response and arrangements of cuts have not been explored, although such a relation, if available, could be useful for designing commercial, engineering, or artistic applications. Here, we performed a systematic study on the force-extension relation for sheets of papers with regularly arranged cuts. As a result, we find a number of regimes for the mechanical response and clarify the physics of the transition between the first rigid and second soft regimes, at the level of scaling laws.

## Experiment

In this study, we focus on simple perforation patterns as shown in Fig. 2(a) and in the Supporting Information video file. Patterns are fabricated by a commercial cutting plotter (silhouette CAMEO, Graphtec Corp). The patterns are characterized by the length *w* of each cut (*w* ≃ 10–30 mm), the horizontal and vertical spacing *d* between the cuts (*d* ≃ 1–5 mm), with *w* and *d* satisfying the condition *w* > *d*. The number *N* of cuts of length *w* is fixed to 10, making the sample height to be 2*Nd* as indicated in Fig. 2(a). The sample can be regarded as a serial connection of 2*N* elementary plates characterized by the lengths, *h*, *d*, and *w* + 2*d* ≃ *w*. The material of sheet samples is Kent paper (high quality paper with fine texture mainly used for drafting) of thickness *h* (*h* ≃ 0.2–0.3 mm), whose Young’s modulus *E* is measured to be in the range *E* ≃ 2.45–3.27 GPa (with the standard deviations less than ≃5%). We measured force as a function of extension by a force gauge (FGP-0.2, NIDEC-Shimpo) mounted on an automatic slider system (EZSM6D040, Oriental Motor) as in the previous studies on fracture^17^,^18^,^19^. The extension speed is fixed to a slow speed (0.5 mm/s) to remove dynamic effects. In order to minimize experimental errors, the data in Figs 3 and 4 below are obtained within a short period in which temperature and humidity are relatively stable.

An example of the overall mechanical response is shown in Fig. 2(b) with snapshots. In the first regime, which is linear as shown in the inset, the deformation is restricted in-plane deformation: small stretch occurs as a result of in-plane bending of elementary plates (a simplified view is shown in Fig. 2(c)). In the second regime, out-of-plane bending of elementary plates accompanied by their rotation of angle *θ* allows more stretch (see Fig. 2(d) for a simplified view). In the third regime, the deformation is rather localized near the tips of the cuts, leading to hardening of the mechanical response and finally to fracture.

## Theory

The principle results of this paper can be summarized as follows. In the initial to second regime, the in-plane deformation energy competes with the out-of-plane deformation energy. The transition between the two regimes occurs when the two energies become equal. This condition is found to be given by the following critical extension 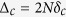 or critical strain 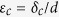:


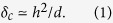


In the initial regime (Δ < Δ_*c*_), the response can be described by the force-extension law or the stress-strain relation, which is linear:


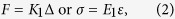


where 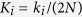 and 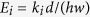 with


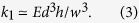


In the second regime (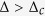), the response becomes quasi-linear:


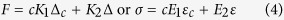


with 0 < *c* < 1 where


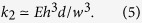


This transition is certainly from hard to soft regime, as confirmed by the ratio 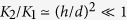. The observed drop of force at the transition can be estimated as (1 − *c*)*K*_1_Δ_*c*_ because 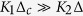 for small *θ*. In the following, these relations are theoretically explained with theoretical limitations and the agreement between theory and experiment is shown.

In order to understand the mechanical response, we remind the bending energy of a plate of length *L*, width *b*, and thickness *a* for the small bending deflection *δ* (*δ* ≪ *L*)^20^:


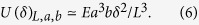


Dimensionally, this is given as follows (see Fig. 2(e)). We set the *x*, *y*, and *z* axes in the direction of *L*, *a*, and *b*, respectively. The energy per unit volume for a bending of the plate (Young’s modulus *E*) characterized by the curvature *R* scales as *Eε*^2^/2 (this is exact when Poisson’s ratio *ν* is zero), where the strain is estimated by *ε* = ((*R* + *y*)*φ* − *Rφ*)/*Rφ* = *y*/*R* with *φ* the central angle of the arc in Fig. 2(e) when the plate occupies the region 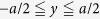. For the deflection *δ* of the plate in the *y* direction, the total bending energy is given by 
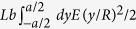
, which leads to Eq. (6) with the numerical coefficient 8/(3(1 − *ν*^2^))^20^, because the radius of curvature 1/*R* is given by 2*δ*/(*L*/2)^2^ in the limit *δ* ≪ *L*.

To characterize the mechanical response in the initial regime, we simply consider superposition of the in-plane bending illustrated in Fig. 2(c). By identifying the parameter set (*L*, *a*, *b*) with the set (*w*, *d*, *h*), we obtain the deformation energy in the initial regime:


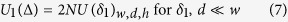


with Δ = 2*Nδ*_1_ because our test samples can be regarded as a serial connection of 2*N* elementary plates. This energy scaling with Δ^2^ results in the linear force-extension relation in Eq. (2) with Eq. (3). Here, the stress *σ* and the corresponding elastic modulus *E*_1_ are introduced by the definitions *σ* = *F*/(*hw*) and *σ* = *E*_1_Δ/(2*Nd*).

The mechanical response in the second regime can be estimated by simply considering superposition of the out-of-plane bending with rotation illustrated in Fig. 2(e). With the replacement (*L*, *a*, *b*) → (*w*, *h*, *d*), the deformation energy is given by


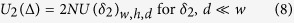


with the relation 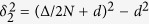 (see the triangle in Fig. 2(d)). The energy in Eq. (8) scaling with (Δ/2*N* + *d*)^2^ − *d*^2^ leads to the quasi-linear force proportional to Δ/2*N* + *d* in Eq. (4) with Eq. (5).

The crossover from the initial to the second regime occurs when the two energies *U*_1_(Δ) and *U*_2_(Δ) coincide with each other, which leads to Eq. (1). For a given Δ, the deformation with the smaller energy is favored, confirming the crossover from *U*_1_(Δ) to *U*_2_(Δ) at Δ = Δ_*c*_ as Δ increases. We can show that the numerical coefficient for Eq. (1) and Eq. (3) are of the order of unity and 0 < *c* < 1 in Eq. (4), as announced, in a naive assumption in which the numerical coefficients for Eq. (7) and Eq. (8) are both given by 8/(3(1 − *ν*^2^)).

## Experiment and theory

Equation (3) for the stiffness constant *K*_1_ can be well confirmed as shown in Fig. 3(a,b). This quantity, experimentally determined from the slope of a plot as shown in the inset of Fig. 2(b), is given as a function of *h* for various *d* and *w* in Fig. 3(a). When the two axes are rescaled according to Eq. (3), namely, 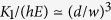, all the data in Fig. 3(a) collapse onto a master curve as shown in Fig. 3(b), confirming the theory. The slight discrepancy that can be recognized for the data 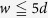 is consistent with the prediction because the theory requires the condition *w* ≫ *d*. This collapse predicts the numerical coefficient for this scaling law to be 0.346 ± 0.006 (based on the data with *w* > 5*d*), which is of the order of unity, as expected.

Equation (1) for *δ*_c_ can also be well confirmed as shown in Fig. 4(a,b). The critical extension Δ_*c*_ can be estimated as the end point of the initial linear regime as shown in the inset of Fig. 2(b). This quantity is given as a function of *h* for various *d* and *w* in Fig. 4(a). When the two axes are rescaled according to Eq. (1), namely, 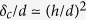, all the data in Fig. 4(a) collapse onto a master curve as shown in Fig. 4(b), confirming the theory. The slight discrepancy recognized for the data with 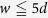 is again consistent with the prediction. According to this collapse (of the data with *w* > 5*d*), the numerical coefficient for the scaling law in Eq. (1) is obtained as 3.02 ± 0.05. This value is of the order of unity as expected.

## Discussion

The assumption employed in Eqs. (7) and (8) that all the elementary plates behave in the same way may be reasonable at the level of scaling laws, as strongly supported by the good agreement between theory and experiment. The extension from this level of description should be examined further in a separate study.

**Figure 1:**
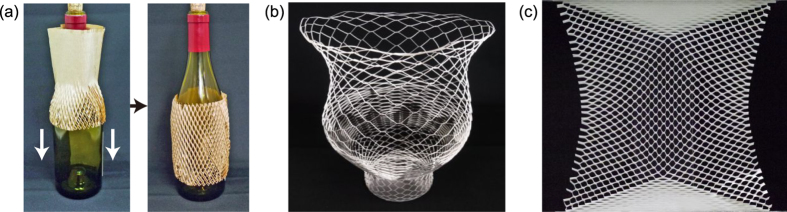
(**a**) A sheet of paper perforated with many cuts, *kirigami*, used for the protection of a bottle of wine. (**b**) “Airvase” (Torafu Architects, Japan) sold in museum shops worldwide, made from a sheet of kirigami. (**c**) Planer stretching of a sheet of kirigami with similar perforation geometry. The lack of circular symmetry leads to inhomogeneously stretched cuts.

**Figure 2:**
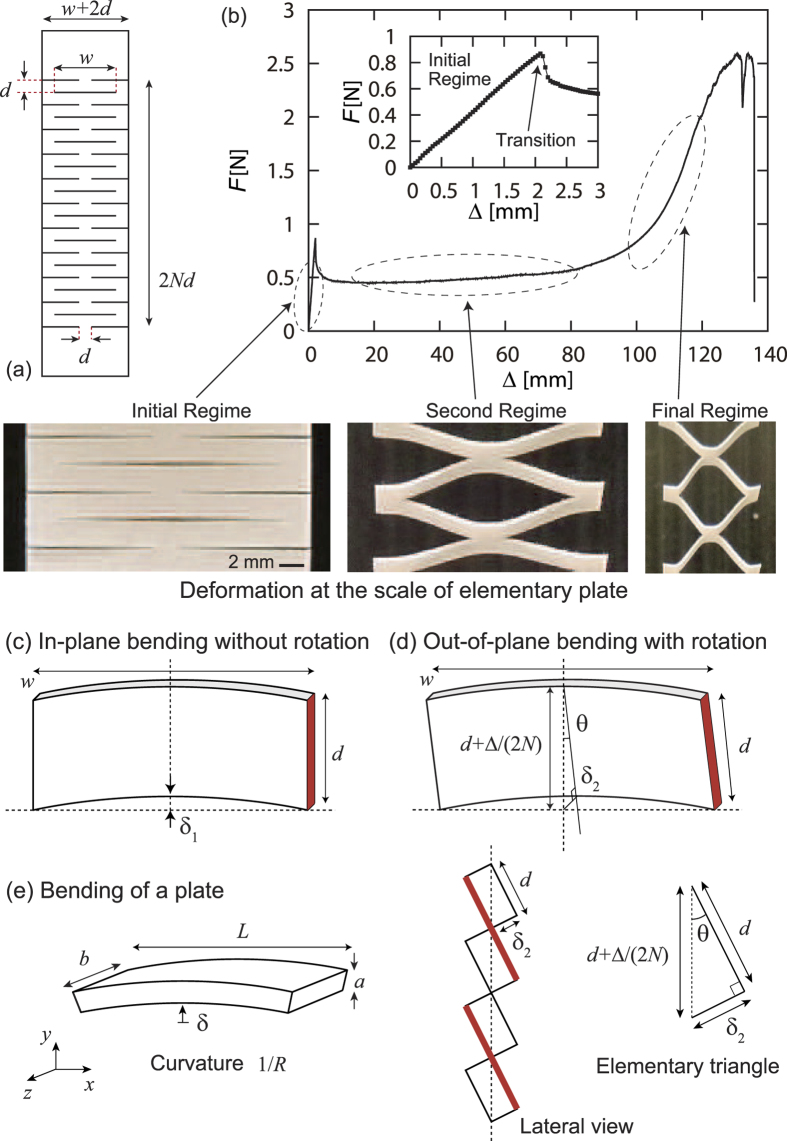
(**a**) Kirigami pattern investigated in the present study. (**b**) Force *F* vs. extension Δ. The initial regime shown in the inset is linear, which is followed by the second soft regime and the final hardening regime. (**c**) In-plane deformation of the unit plate in the initial regime. (**d**) Out-of-plane deformation in the second regime: perspective view in the top and lateral view in the left bottom. (**e**) Illustration of bending of a plate to discuss the deformation energy.

**Figure 3:**
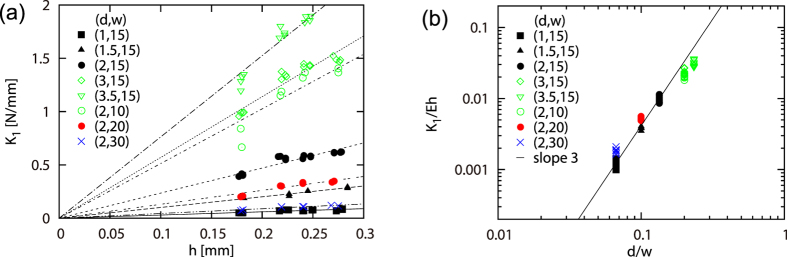
(**a**) Stiffness constant *K*_1_ vs thickness *h* for various cut length *w* and spacing *d*. The lines are guide for the eyes. (**b**) *K*_1_/(*hE*) vs (*d*/*w*)^3^ demonstrating collapse of the data in (**a**) by rescaling of the both axes in (**a**) according to Eq. (3). The slight deviation of the open (green) symbols is consistent with the prediction: for these data the condition 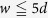 is satisfied whereas the theory requires the condition *d* ≪ *w*.

**Figure 4:**
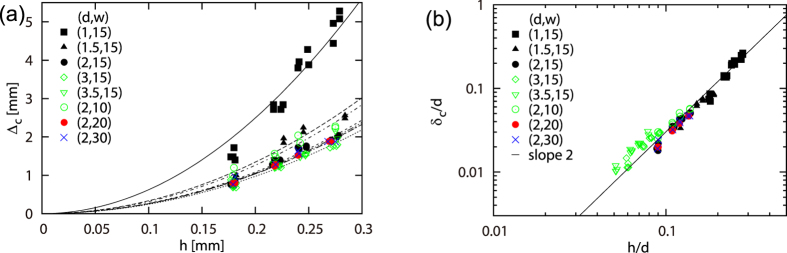
(**a**) Critical spacing Δ_*c*_ vs thickness *h* for various length *w* and spacing *d*. The curves are guide for the eyes. (**b**) *δ*_*c*_/*d* vs. (*h*/*d*)^2^ demonstrating collapse of the data in (**a**) by rescaling of the both axes in (**a**) according to Eq. (1). The slight deviation of the open (green) symbols is again consistent with the prediction.

Although no previous studies are available that focus on the initial rigid regime and the transition of this regime to the following softer regime, at the time of writing this paper we find a number of recent related studies mainly in the engineering community as mentioned the introductory paragraph (In fact, we started the present study, inspired by our previous study^21^ and examples in Fig. 1). Observations in the previous studies are qualitatively explicable by our simple theory: (1) The observations and finite-element-modeling (FEM) calculations in the previous study^13^ focusing on the second regime are consistent with Eq. (5), confirming that the soft spring constant increases as *d* increases and as *w* decreases. (2) In molecular-dynamics (MD) simulations performed for a graphen kirigami in the previous study^15^, the initial rigid regime is practically not observed, which is consistent with our prediction of the disappearance of the initial regime in the limit *h* ≪ *d* (see below).

Our results provide guiding principles in the form of simple scaling laws to control and design similar kirigami structures. The control and design are important from two opposite aspects. (1) One aspect is to realize stretchable sheet materials from stiff materials. In such a case, this initial regime is unfavorable and Eq. (1) gives a clear principle to reduce the range of this initial regime: this regime disappears in the limit *h* ≪ *d*. This prediction is consistent with the previous study^15^ as mentioned above. (2) The opposite aspect is positive utilization of the initial rigid regime, for which we propose two new directions of applications (Note, however, that even in the “rigid regime” the kirigami sheet is already significantly soft compared with the original material as seen from the factor (*d*/*w*)^3^ in Equation (3)). One possibility is fine tuning of the elastic constant of sheet materials. By virtue of Eq. (3), we could design the elasticity of sheet materials at will (in the initial regime). It would be interesting, for example, to use relatively thick sheet materials, widening the range of the initial regime. Another possibility is the application for mechanical force sensors. Because of the sudden elongation at the critical length and force, we could design force sensors on the basis of Eq. (1) with Eq. (2). In addition, Eq. (5) for small deformation should be useful to design and control the soft response of the kirigami sheets (note that Equation (5) qualitatively justify observations in the previous study^13^ as mentioned above). A promising example of applications relevant to the present study would be the use of the kirigami structure for cell sheets, which have received considerable attention in regenerative medicine^22^.

As pointed out already in the above, our prediction is quite consistent with the FEM results in the previous study^13^, whereas the FEM approach and the model proposed here have advantages and disadvantages. The FEM approach predicts the results numerically, whereas the present model predicts the results analytically but without a precise prediction for a numerical coefficient. The numerical coefficient is precisely determined only through a comparison with experimental data. But once this is done, the present model predicts the results numerically in a wide range of important physical parameters without any technical efforts required for the FEM approach. In addition, the present approach provides physical insights into the phenomenon in a clearer manner, giving simple guiding principles for designing the kirigami structure. However, the present prediction can be used only for the cases satisfying the required conditions, such as *d* ≪ *w*, unlike the FEM analysis; the distribution of strain and stress is only available in the FEM analysis.

We consider that the nonlinearity in the stress-strain relationship of sheet materials may not strongly affect the physical pictures provided in the present study. Most of materials certainly possess such nonlinearity for large stresses, whereas the kirigami structure contains many cuts at the tips of which stress could be high. Thus, it would be natural to ask how such nonlinear effects affect the present framework. This problem could be in general nontrivial. However, we expect that nonlinear effects tend to be suppressed in the kirigami structure at least in the initial and second regime, which are the focus of the present study. This is because of the following reasons: (1) In the initial regime the deformation is generally very small. (2) In the second regime the apparent deformation is large but the basic mode of deformation is still bending, whereas bending is intrinsically related to small deformation especially when the plate is thin. This expectation for a non-significant role of the nonlinearity is supported by the agreement between theory and experiment in the present study. However, we experimentally observed that the linearity of the slope in the initial regime could be slightly deteriorated near the transition point in certain cases (although such data points can be well explained by our theory). This might be a slight effect of the nonlinearity in the stress-strain relationship of the sheet material.

## Conclusion

We investigated the mechanical response of a simple and representative kirigami structure that remarkably changes original mechanical properties in a systematic way. As a result, we found simple scaling laws that govern the stiffness of the initial regime and the consecutive softening transition. This transition was revealed to be a transition from the two-dimensional to three-dimensional deformation, i.e., from stretching to bending. The result obtained here could be useful as design principles for simple kirigami structures. Upon seeing the recent surge of engineering utilization of kirigami structures, we envision that the present results would be useful for various applications, as well as for fundamental understanding of the mechanics of sheet materials.

## Additional Information

**How to cite this article**: Isobe, M. and Okumura, K. Initial rigid response and softening transition of highly stretchable kirigami sheet materials. *Sci. Rep.* **6**, 24758; doi: 10.1038/srep24758 (2016).

## Supplementary Material

Supplementary Video 1
